# Antimalarial activity of extract and fractions of *Castanopsis costata* (Blume) A.DC

**Published:** 2019

**Authors:** Maulana Yusuf Alkandahri, Afiat Berbudi, Novi Vicahyani Utami, Anas Subarnas

**Affiliations:** 1 *Department of Pharmacology and Clinical Pharmacy, Faculty of Pharmacy, Padjadjaran University, Jatinangor, West Java, Indonesia.*; 2 *Department of Biomedical Sciences, Parasitology Division, Faculty of Medicine, Padjadjaran University, Bandung, West Java, Indonesia.*; 3 *Department of Biomedical Sciences, Pharmacology Division, Faculty of Medicine, Padjadjaran University, Bandung, West Java, Indonesia.*; † *Equal first author*

**Keywords:** Malaria, Castanopsis costata Antimalarial drugs, Plasmodium berghei

## Abstract

**Objective:**

One of the biggest health problems in the world, which occurs in more than 90 countries, is the spread of malaria. Cep-cepan leaves (*Castanopsis costata*), was empirically used as an antimalarial herb in North Sumatra. Since its use has not been scientifically studied, we investigated the antimalarial activity of extract and fractions of *C. costata* against *Plasmodium berghei* ANKA *(PbA)* in a mouse model.

**Materials and Methods:**

This experimental study was conducted using 32 male Balb/C mice. PbA inoculation was performed intraperitoneally with 10^6^ parasites/mouse. Immediately after parasitemia reach >2% (day 0), the mice were treated orally with daily artesunate (36.4 mg/kg/day) (positive control), ethanolic extract (100, 200, and 400 mg/kg/day), and the fractions of water, ethyl acetate and n-hexane (108 mg/kg/day each) for 5 consecutive days (from day 0 to 4). Parasitemia inhibition was observed to determine the antimalarial activity of each type of *C. costata* extract and fractions.

**Results:**

The administration of *C. costata* leaves ethanolic extract (100, 200, and 400 mg/kg) significantly inhibited the growth of *PbA *in Balb/C mice (42.66%, 66.2 1% and 80.99 % inhibition, respectively) (p<0.05). Similarly, all *C. costata* fractions also produced antimalarial activity against *PbA* with administration of the ethyl acetate fraction presenting the highest activity (79.85 % inhibition).

**Conclusion:**

The *C. costata* leaves showed antimalarial activity against *PbA*. However, further studies are necessary to elucidate the underlying mechanisms of this effect and the active compounds involved. Our current study revealed that *C.costata* could be a potential candidate to be used as a new antimalarial drug.

## Introduction

Malaria remains the main cause of death in developing countries with tropical climates. It was estimated that around 350-500 million people in the world were infected with this disease and more than 1.5 million people die each year due to malaria. Rapid population growth and overpopulation, migration, and poor sanitation, have increased the spread of the disease. Malaria becomes a serious threat since many studies reported parasitic resistance upon the first-line antimalarial drugs used in the treatment and prevention of malaria (Burke et al., 2003 ▶; Hayati et al., 2012 ▶). Given that no effective antimalarial drugs have been found so far, the efforts to introduce new antimalarial drugs, both synthetic drug and plant-based medicine, are still on going, especially in exploration of plants that were traditionally used to treat malaria in various endemic regions of the world (Mustofa, 2003 ▶). Natural products particularly those used in the plant-based medicine, contain a great variety of chemical structures. Many studies screened the antiplasmodial activities of some plants as potential sources of new antimalarial drugs. Ethnopharmacological approaches seem to be promising in finding new antimalarial candidates (Bero and Quetin-Leclercq, 2011 ▶; Nogueira and Lopes, 2011 ▶; Saxena et al., 2003 ▶).

Indonesia is the second country with the the largest biodiversity in the world with the forests where 28.000 species of plants are found. It was estimated that around 2,500 plants species have potential medicinal efficacy (Elfahmi et al., 2014 ▶). Cep-cepan plant (*Castanopsis costata *(Blume) A.DC.) is one of plants that were used empiricaly as an antimalarial drug by the people of North Sumatra, Indonesia. However, the administration of *C. costata* as an anti-malaria medication has not been scientifically investigated. As a member of Fagaceae family, *C. costata* has been known to have several therapeutic properties, such as antifever, digestion disorder relieving, wound healing, and pain killing effects (Salim et al., 2017 ▶). Previous studies reported that *C. costata* has a variety of pharmacological activities such as antibacterial (Nurtjahja et al., 2013 ▶), antioxidant (Alkandahri et al., 2016 ▶), analgesic (Salim et al., 2017 ▶), and anti-inflammation effects (Alkandahri et al., 2018 ▶). While the anti-malaria effect of *C. Costata* has not been studied yet, its close family called *Quercus laceyi* was reported to have antimalarial activity against *Plasmodium falciparum* (Cai et al., 2016 ▶; Pan et al., 2018 ▶). 

Since *C. costata* has similarities with *Q. lacecy* that is known for its antimalarial activity, we investigated the antimalarial activity of *C. costata* in experimental animals infected with *Plasmodium berghei* ANKA (*PbA*).

## Materials and Methods

All of the experiments in this study were performed in Pharmacology and Toxicology Laboratory, Faculty of Pharmacy, Padjadjaran University, Bandung, Indonesia. The experimental animal works have been approved by the Research Ethics Committee of Padjadjaran University Bandung (No. 507/UN6.KEP/EC/2018) and were exactly conducted according to the “Principles of Laboratory Animal Care” (NIH Publication No. 86-23).

**Plant determination**

Fresh leaves (10 kg) of *C. costata *were collected from Namu Keling Village, North Sumatera and transported to Phytochemistry and Pharmacology Laboratory, Padjadjaran University, for cleaning, air drying, milling, and extraction process. The plants were then identified as *C.costata* by Herbarium unit, Department of Biology, Faculty of Mathematics and Science, Padjadjaran University (Code: 219/HB/04/2017).

***C. costata*** **extraction and** **fractionation**

The *C. costata* leaves powder (1 kg) was exhaustively macerated in 70% ethanol for 72 hr. The liquid extract was obtained and concentrated using rotary evaporator at 50^o^C to produce around 9.87% concentrate (fixed weights of the extracts were divided by simplicia weights multiplied by 100%). The extract was stored at 4^o^C before being used for the experiment. The dried ethanolic extract was dissolved in distilled water for preparing various doses. After dissolved in an ethanol-water (1:3) mixture, the ethanol extract of *C. costata* leaves (50 g) was fractionated by liquid–liquid partition, using ethyl acetate (EA) (4 × 150 ml), and hexane (4 × 150 ml). This procedure produced three fractions including *n*-hexane (16.54 g, 33.08%), ethyl acetate (EA) (19.68 g, 39.36%) and water (10.17 g, 20.34%).

**Phytochemical screening test**

The extract and fractions of *C. costata* leaves were qualitatively screened to identify the presence of secondary metabolites including alkaloids, flavonoids, terpenoids, phenolic compounds, tannins, saponins, and anthraquinones glycosides (Yadav and Agarwala, 2011 ▶).

**A** **nimals**

Thirty two male Balb/C (25-30 g and 8-12 weeks old) were obtained from animal house of School of Pharmacy, Bandung Institute of Technology (Bandung, Indonesia) and housed under standard conditions with 12 hr-12 hr light-dark cycles. Four mice were housed in each cage and they had free access to food and water *ad libitum*. In order to acclimatize to the environment, the animals were transferred to laboratory 7 days before initiation of the experiments. Animal procedures were performed according to guidelines for the care of experimental animals of Faculty of Medicine, Padjadjaran University.

**Parasite inoculation**

Animals were infected with *PbA* by injecting each mouse with 0.2 ml of *PbA*-infected blood (1×10^6^ *PbA* parasitized red blood cells) intraperitoneally, and parasitemia levels was then observed daily. Day-0 was defined when parasitemia levels reach at least >2%.

**Drug administration**

Depending on the group, the antimalarial standard drug (artesunate), *C. costata* extract and *C. costata* fractions were administered directly into the mice stomach via oral gavage technique. All treatments were started when parasitemia >2% (day 0) and performed once a day for 5 consecutive days (day 0 to day 4).

**Evaluation of** ***in vivo*** **antimalarial activity of extract and fraction** **s** **of** ***C. costata*** **leaves (4-day test)**

Antimalarial activity of the extract and fractions of *C. costata* leaves was evaluated using the method described by Knight and Peters (Knight and Peters, 1980 ▶). The animals were divided into eight groups. Briefly, after having >2% parasitemia (day 0), the mice were treated with 100, 200 and 400 mg/kg/day doses of the *C. costata* leaves extract, 108 mg/kg/day dose of the *C. costata* leaves fractions (n-hexane, ethyl acetate, water), 36.4 mg/kg/day of artesunate (Guilin Pharmaceutical Co., Ltd) and 1% PGA (Pulvis Gummi Arabicum) suspension (control) for five consecutive days (day 0 to 4). Thin blood smears were made from the tail blood of each mouse every day for 5 days (day 0 until day 4) and stained using 10% giemsa to observe the infected-red blood cells under microscope. The average of parasitemia and % parasitemia inhibition were calculated and expressed using the following formula (Fidock et al., 2004 ▶).

% Parasitemia = Total number of parasitized red blood cells1000 red blood cells x 100%

% Parasitemia inhibition = Parasitemia in control group - Parasitemia in study groupParasitemia in control group x 100%

**Microscopic observation**

 Parasitemia levels of thin blood smear of all mice were analyzed under the microscope (Nikon Eclipse E100, Japan) with 1000x magnification. In addition, the morphology of each growth phase of P. berghei ANKA in host red blood cells were also observed.

**Statistical analysis**

Data were analyzed using SPSS software (version 22). For statistical analysis, one way analysis of variance (ANOVA) followed by Tukey’s was applied. The results are presented as mean±S.E.M and p-values less than 0.05 were considered significant.

## Results

**Phytochemical screening**

The phytochemical screening of *C. costata* extract revealed the presence of chemical constituents such as phenolics, alkaloids, flavonoids, saponins, tannins, triterpenes, and anthraquinone glycosides, whereas the *C. costata* leaves water fraction contained phenolics, flavonoids, saponins, and tannins. Meanwhile, ethyl acetate fraction of *C. costata* leaves showed the presence of phenolics, flavonoids, saponins, tannins, and triterpenes. Furthermore, n-hexane fraction of *C. costata* leaves contained alkaloids and triterpenes. A summary of the phytochemical screening of extract and fractions of *C. costata* leaves is presented in Table 1.

**Table 1:** Phytochemical screening of extract and fractions of *C. costata* leaves

**Phytochemical screening**	**Extract**	**Water fraction **	**Ethyl acetate fraction **	***n*** **-Hexane fraction **
**Phenolics**	+	+	+	-
**Alkaloids**	+	-	-	+
**Flavonoids**	+	+	+	-
**Saponins**	+	+	+	-
**Tannins**	+	+	+	-
**Triterpenes**	+	-	+	+
**Anthraquinone glycosides**	+	-	-	-

(+)=Contained, (-)=Not contained

**Antimalarial activity**

**Parasitemia observation**

**Figure 1:**
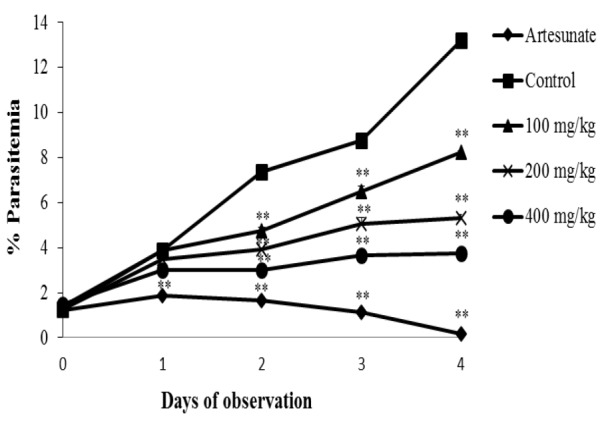
*In vivo* antimalaria activity of extract of *C. costata*. The graph represents % parasitemia upon administration of drug (artesunate), PGA (control) and extract. Data are presented as mean±SEM of four animals in each group. ** shows p<0.05 compared to the control group. PGA: Pulvis Gummi Arabicum

**Figure 2:**
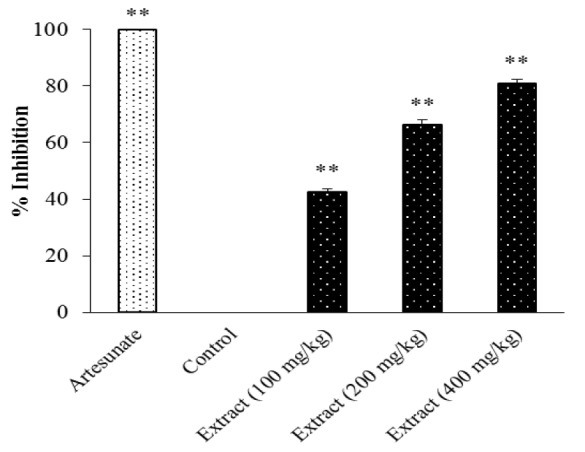
*In vivo* antimalaria activity of *C. Costata *fractions. The graph represents % parasitemia upon administration of drug (artesunate), PGA (control) and fractions. Data are presented as mean±SEM of four animals in each group. ** shows p<0.05 compared to the control group. PGA: Pulvis Gummi Arabicum

Our observation on day 0 to 4 revealed that both mice groups treated with ethanolic extract (Figure 1) and fractions of *C. costata* (Figure 2) showed lower increases in parasitemia compared to the control group, indicating that administration of ethanolic extract and fractions of *C. costata* inhibited *P. berghei* development in mice. Although progressive inhibition of parasitemia was observed, this effect was still below parasitemia inhibition produced by artesunate (Figures 1 and 2).

**The e** **ffect of extract and fractions of** ***C. costata*** **leaves** **on** ***PbA-*infected mice during 4-day Peter’s suppression test**

**Figure 3:**
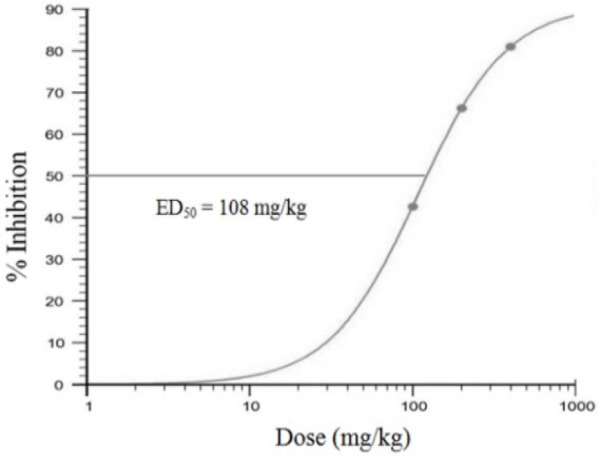
The anti-malaria activity of *C. costata* leaves extract against *PbA* in mice. Three doses of extract (100, 200 and 400 mg/kg) were given orally daily for 4 days. Artesunate (36.4 mg/kg, p.o) was used as a standard drug. Data are presented as mean±SEM of four animals in each group. ** shows p<0.05 compared to the control group

Figure 3 shows that the parasitic inhibition effect of the extract of *C. costata* leaves was dependent on the administered dose. The administration of *C. costata* extracts (100, 200 and 400 mg/kg) inhibited parasitemia (p<0.05), but their impacts were lower than that of artesunate. The higher doses showed stronger parasitemia inhibition. 

**Figure 4:**
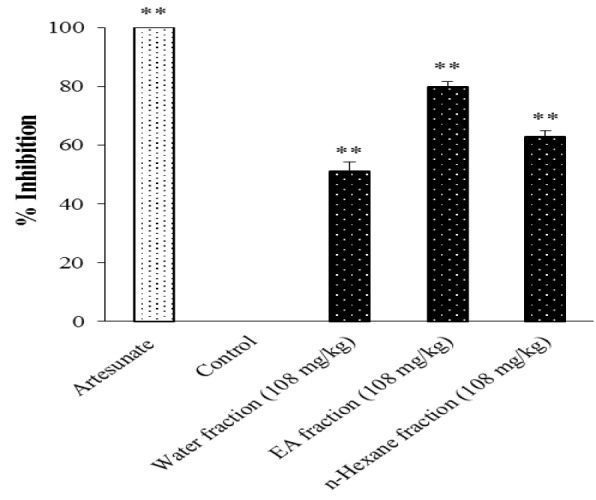
Dose-response effect of extract of* C. costata *leaves

From the results of the probit analysis, it was found that an effective dose (ED50) to inhibit parasite growth was reached upon oral administration of 108 mg/kg/day C. costata extract (Figure 4).

**Figure 5:**
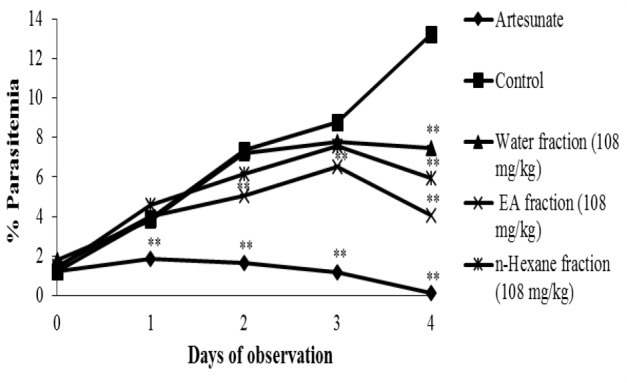
The anti-malaria activity of fractions of *C. costata* leaves in *PbA*-infected mice. Three fractions (water, ethyl acetate and *n*-hexane) (108 mg/kg) were given orally daily for 4 days. Artesunate (36.4 mg/kg, p.o) was used as a standard drug. Data are presented as mean±SEM of four animals in each group. ** shows *p*<0.05 compared to the control group

The administration of 108 mg/kg/day *C. costata* fractions inhibited the parasitic growth. Its inhibitory effect was dependent on the type of the administered fraction. There was higher parasite growth inhibition upon the administration of fractions/artesunate compared to the control group (p<0.05) (Figure 5), but the effects of the three *C. costata* leaf fractions (108 mg/kg) were less than the effect produced by artesunate.

**Figure 6:**
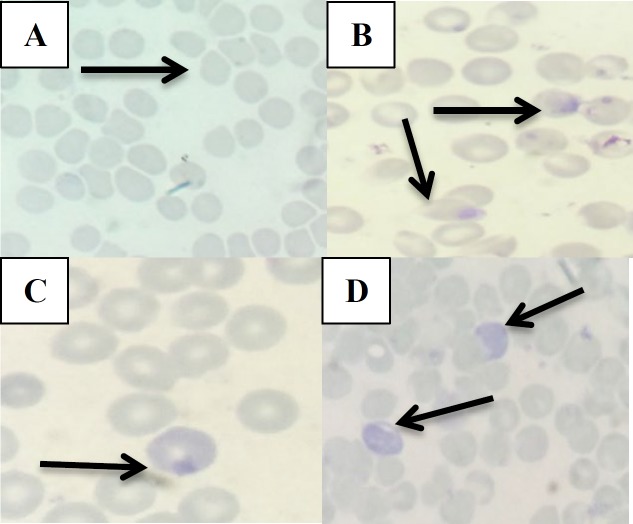
Morphology of each growth phase of PbA for 4 days under a microscope with a magnification of 1000X. A) Normal red blood cells; B) Ring phase; C) Trophozoite phase; D) Schizont phase

Parasites that infect red blood cells produce several changes including enlargement of red blood cells, alteration of the color of red blood cells and the appearance of spots on certain staining. (Cooke et al., 2001 ▶). There are differences in parasite morphology among the groups during 4 days of testing. Morphological differences among the test groups can be seen from the parasitic shape and the color of infected red blood cells (Figure 6). 

## Discussion

Our results revealed antimalarial activity of *C. costata* leaves extract and fractions in *PbA*-infected mice during 5 days administration. These results also indicated that extracts and fractions of *C. costata* leaves are able to inhibit parasite growth in mice. The ethanolic extract of *C. costata* leaves was significantly active against *PbA *in a dose-dependent manner with the inhibitory activity observed in the *C. costata*-treated mice at doses 100 mg/kg (% inhibition: 42.66%), 200 mg/kg (% inhibition: 66.2 1%) and 400 mg/kg (% inhibition: 80,99 %) of the extract (Figure 3). 

Since the administration of *C. costata* leaves extract resulted in >50% parasitemia suppression at doses of 500, 250, and 100 mg/kg body weight, *C. costata* can be classified as an agent with very good anti-malarial activity (Bantie et al., 2014 ▶). Meanwhile, the ethyl acetate fraction of *C. costata* leaves also showed higher parasitic growth inhibition (% inhibition: 79.85 %) if compared to the water fraction (% inhibition: 51.19 %) and n-hexane fraction (% inhibition: 62.77 %) (Figure 5).

The anti-malarial activity of the extract and fractions of *C. costata* leaves could be due to the presence of several secondary metabolite compounds in extract and fractions of the leaves of *C. costata*, especially alkaloid compounds, flavonoids, triterpenoids, phenolics, tannins, saponins, and anthraquinone glycosides. Some secondary metabolites were proven to have anti-malarial activities. Some alkaloids have been known to inhibit parasite growth by inhibiting parasite growth through intracellular choline transport in the blood schizont phase (Hilou et al., 2006 ▶). In addition, seskuiterpenoid and triterpenoid can inhibit parasitic growth by inhibiting heme polymerization into hemozoin through free radical formation of sesquiterpenic lactone which will alkylate heme to form hemartemisinin complexes. Moreover, seskuiterpenoid and triterpenoid have been known are able to inhibit the respiration process in mitochondria, protein synthesis in parasitic cells and Ca^2+^ ion transporters called PfATP6, that is a sarcoendoplasmic reticulum of calcium-dependent ATPases (SERCAs) which is only found in *P. falciparum* (Meschnick et al., 1996 ▶; Pouplin et al., 2007 ▶). Furthermore, flavonoid compounds were also reported to inhibit the growth of malaria parasites by inhibiting the nutrient transport required by the parasite in the intraeritrocytic stage (i.e. blood schizont) (Kirk, 2001 ▶; Kirk, 2004 ▶) and inhibit hemoglobin degradation and heme detoxification in food vacuoles of malaria parasites (Biagini et al., 2003 ▶; Bilia, et al., 2006 ▶; Frolich et al., 2005 ▶).

Phenolic compounds and tannins in the extracts and fractions of *C. costata* leaves are also expected to play a role in inhibiting parasite growth via their antioxidant actions (Kim et al., 2003 ▶). The antioxidant activities of phenolic and tannin compounds are mainly caused by the redox nature of these compounds, which can play an important role in absorbing and neutralizing free radicals, and decomposing peroxide compounds (Laphookhieo et al., 2009 ▶). Furthermore, antioxidant activity of phenolic compounds and tannins can inhibit parasite protein synthesis and also neutralize oxidative damage induced by malaria parasites (Builders et al., 2010 ▶). 

Our study proved that the extract and fractions of the leaves of *C. costata* have the ability to inhibit the growth of malaria parasites in Balb/C mice infected with *PbA*. This suggests that *C. costata* can be potentially used to develop new agents to combat malaria. However, further studies are required to elucidate the mechanisms behind such effects. 
